# Resilience of Juvenile Freshwater Pearl Mussels to Thermal Stress

**DOI:** 10.1002/ece3.70456

**Published:** 2024-10-23

**Authors:** Sebastian Wacker, Katrine Åmdal Sundt, Jon Hamner Mageroy, Bjørn Mejdell Larsen, Chavindi Sophie Hagen, Torill Horvli, Grethe Robertsen

**Affiliations:** ^1^ Norwegian Institute for Nature Research Trondheim Norway; ^2^ Department of Biological Sciences University of Bergen Bergen Norway

**Keywords:** climate change, freshwater mussels, *Margaritifera margaritifera*, survival, temperature

## Abstract

Climate change poses a significant threat to freshwater ecosystems by causing increases of average water temperatures, and more frequent and extreme heating events. Freshwater mussels are declining globally, and the distribution of the freshwater pearl mussel (*Margeritifera margeritifera*) has decreased dramatically over the past century. Even though it is likely that climate change is contributing to the decline of the species, little is known about the specific mechanisms involved. Here, we test how short episodes of water temperatures above the known thermotolerance range affect the survival and growth of the early post parasitic juvenile phase of freshwater pearl mussels. We also test if previous experience with elevated water temperatures can modify survival and growth responses to subsequent high‐temperatures exposure. Mortality was very low in all treatments (< 5%) and not affected by the temperature treatments, while growth rate was positively affected by temperature. Our results suggest that juvenile mussels can survive short periods of heat stress when other environmental conditions are favourable. Future studies should therefore address how heat stress affects survival in combination with other stressors, such as reduced availability of dissolved oxygen.

## Graphical abstract

Results from our experiment suggest that juvenile mussels can survive short periods of heat stress when other environmental conditions are favourable. Future studies should therefore address how heat stress affects survival in combination with other stressors, such as reduced availability of dissolved oxygen.

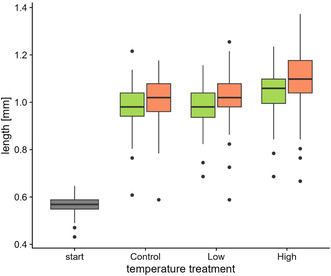

## 1 Introduction

Freshwater ecosystems are currently under threat from a wide range of anthropogenic stressors, and the observed rates of biodiversity loss in freshwater ecosystems far exceed those in terrestrial ecosystems (Heino, Virkkala, and Toivonen 2009; Tickner et al. 2020). Major anthropogenic stressors include land use changes, habitat fragmentation, pollution, and invasive species. In addition, freshwater ecosystems are under peril due to global warming (Heino, Virkkala, and Toivonen 2009; Capon, Stewart‐Koster, and Bunn 2021). One of the gravest consequences of global warming for freshwater ecosystems are rising water temperatures, which are directly linked to elevated air temperatures. Global warming may not only lead to rising average temperatures, but also to more frequent and pronounced extreme climate events (IPCC 2021). More frequent and intense heat waves and drought periods will result in higher maximum water temperatures. Periods of extreme temperatures are considered a major threat to freshwater organisms, which often are ectotherms (i.e., with a body temperature very close to the temperature of the surrounding) with limited mobility (Heino, Virkkala, and Toivonen 2009; Capon, Stewart‐Koster, and Bunn 2021).

The freshwater pearl mussel (*Margeritifera margeritifera*) inhabits oligotrophic rivers in Europe and North America. Freshwater pearl mussels are very long‐lived (up to 250 years) and have a complex life cycle, involving a parasitic stage spent on the gills of salmonid fishes (Karlsson, Larsen, and Hindar 2014; Lopes‐Lima et al. 2017; Salonen et al. 2017). Larvae (glochidia) released by female mussels attach to the gills of the fish host, where they develop over a period of 9–11 months, before they detach and further develop for a period of at least 5–8 years in the riverbed substratum (Bauer 1987; Hastie and Young 2003; Larsen 2018). The mussels then emerge from the riverbed substratum and mature. The species historically inhabited large parts of Europe, but its distribution range has decreased dramatically over the past century (Lopes‐Lima et al. 2017) and it is now listed as endangered in the IUCN red list (IUCN 2017). Freshwater pearl mussel populations are threatened by a wide range of anthropogenic factors, including habitat degradation, harvesting, pollution and decreasing host fish populations. Habitat degradation often involves siltation and sedimentation that lead to loss of riverbed substratum that is sufficiently oxygenated for the development of juvenile mussels (Buddensiek et al. 1993; Hastie, Boon, and Young 2000; Geist and Auerswald 2007). Conservation programmes for the species are put in place in many European countries and involve habitat restauration and captive breeding programmes (Gum et al. 2011, Ferreira‐Rodriguez et al. 2019; Geist et al. 2023).

Global warming is considered a major threat to the remaining freshwater pearl mussel populations in Europe (Hastie et al. 2003; Santos et al. 2015; Bolotov et al. 2018). Increased water temperatures are likely to affect all life‐stages of freshwater pearl mussels. While modest increases in average water temperatures may benefit mussel populations in some regions, by inducing faster growth (Hruska 1992; Cerna et al. 2018), negative effects are expected across large parts of the species distribution, due to extreme temperatures that exceed the species' thermotolerance range (Hastie et al. 2003; Bolotov et al. 2018).

Most of the viable (recruiting) freshwater pearl mussel populations are today found in Fennoscandia, at the northern range of its species distribution, where water temperatures tend to be lower. At the southern range of its distribution, viable populations are limited to high altitude habitats (Santos et al. 2015). More than 95% of the remaining populations in central and southern Europe lack recruitment and are considered functionally extinct (Young, Cosgrove, and Hastie 2001; Geist 2010). Norway holds a large proportion of the remaining viable populations, but recent analysis of over 300 populations showed a negative relationship between average summer temperature and the likelihood of recruitment (Gosselin et al. 2023). A recent study, that used shell morphology to infer temperature thresholds for the species, concluded that global warming may dramatically reduce the suitable habitat in Europe, which under extreme climate change scenarios may be restricted to higher elevation areas of Fennoscandia, the UK and Ireland (Bolotov et al. 2018).

Despite growing evidence that rising water temperatures can have negative effects on freshwater pearl mussels, little is known about the mechanisms involved, and experimental tests of the species' thermotolerance are lacking. This is unfortunate because an understanding about the mechanisms, and at what life stages and time periods the freshwater pearl mussels are most sensitive to increased temperatures, is key to design mitigation measures necessary to protect the species. Also, little is currently known about the effects of environmental stressors in the initial period after juveniles detach from their host to take up a sedentary life. In the juvenile phase freshwater pearl mussels live buried in the substratum and cannot escape detrimental water temperatures under extreme climate events. This early post‐parasitic stage is known to be sensitive to environmental stressors, such as increased eutrophication and reduced oxygen levels (Hyvärinen et al. 2021). The contribution of temperature extremes to mortality of juvenile mussels is however still largely unknown.

In this study, we use a controlled experimental set‐up to test how short episodes of water temperatures above the known thermotolerance range (0°C–26°C, Jungbluth and Lehmann 1976) affect the survival and growth of the early post parasitic juvenile phase of freshwater pearl mussels. Thermotolerance may be modified by previously experienced temperatures, and individuals exposed to heat stress may obtain a larger thermotolerance through acclimatisation (Moyen et al. 2020). We therefore compare survival and growth of juvenile mussels exposed to heat stress after a previous high‐temperature episode with those kept under standard temperatures.

## 2 Methods

### 2.1 Production and Husbandry of Juvenile Mussels

Experiments were carried out at the rearing station for freshwater pearl mussels (FPM) in Norway, at Austevoll (Vestland County). Production and husbandry of juvenile mussels followed standard protocols at the rearing station (Marwaha et al. 2017, 2019, 2021). Adult FPM (*N* = 60) were collected from River Etna (Innlandet County) after fertilisation had taken place and transferred to a 1 × 1 m tank with permanent water flow (25–35 cm/s). Natural recruitment had been absent in the population in previous years and the population was classified as vulnerable according to the Norwegian classification system for the conservation status of FPM (Larsen and Magerøy 2019). When mussels started to release larvae, glochidial strings were collected and used to infest naïve hatchery‐reared 0+ age brown trout. Brown trout is the natural host of FPM in River Etna (Larsen 2000). Infested brown trout were kept in holding tanks until juvenile mussels started to detach. Fish were then transferred to collection chambers, where detaching juveniles were collected with 200 μm sieves. Sieves were inspected daily, juveniles cleaned from debris and transferred to plastic boxes (280 × 190 × 140 mm) for further husbandry. All juveniles used in the experiment were collected on 28 June 2021.

**FIGURE 1:**
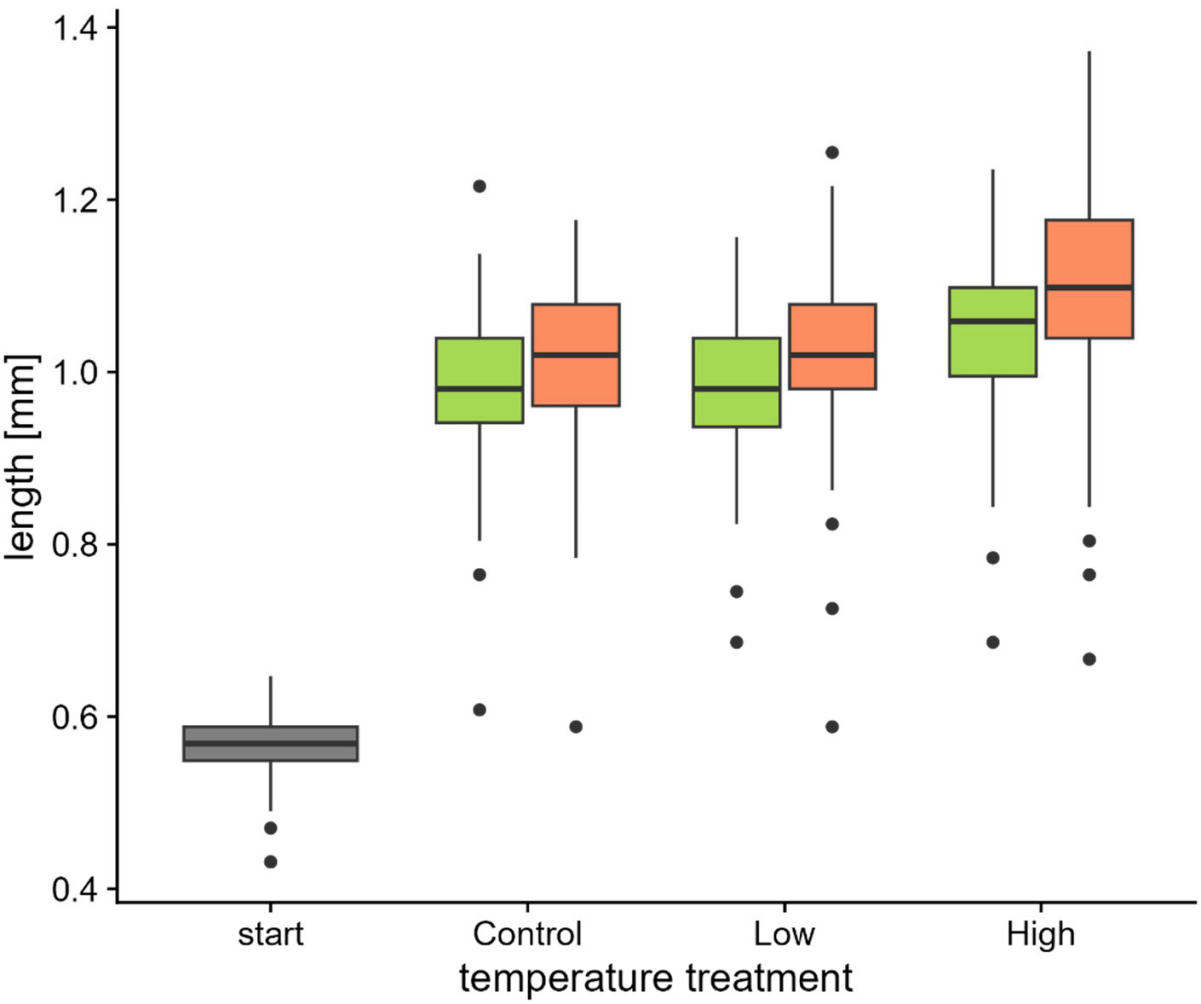
Length of juvenile freshwater pearl mussels at the start of the experiment (*start*) and after exposure to a first (*control*, *low*, *high*), and second round of temperature treatment (green boxplots: Control temperature, red boxplots: High temperature).

Boxes with juvenile mussels were kept in a temperature‐controlled room at 17.0°C prior to the experiment. Juveniles were fed two times a week with a solution containing Shellfish diet 1800, Nanno 3600 (Reed Mariculture Inc., Campbell, CA, USA), and cultures of *Scenedesmus* sp. and *Nannochloropsis* sp. (NORCCA, Oslo, Norway) (Marwaha et al. 2017). Feeding involved the exchange of water, during which the old water was removed and replaced with fresh water containing a mixture of feeding solution and detritus. Detritus was collected once a week in a nearby swamp and kept under oxygen‐rich conditions until use. Water was supplied from a nearby lake (Lake Kvernavatnet), filtered and treated with ozone 3 days before use (Marwaha et al. 2017). Exchange‐water was pre‐adjusted to the temperature of the respective treatment. We started the experiment on 12 July, 14 days after the excystment of juvenile mussels. Juveniles had an average size of 0.56 mm (SD: 0.04 mm, *N* = 80) at the start of the experiment (Figure 1; Appendix S3).

### 2.2 Experimental Treatments

The first part of the experiment (Days 1–28) consisted of two treatments of increased water temperature (target maximum temperatures 23.0°C and 26.0°C) and one control treatment (18.0°C, which was standard rearing temperature). Ten replicates of each treatment were run in parallel in individual boxes and each replicate contained 30 juvenile mussels. In the second part of the experiment (Days 29–40), juveniles from half of the replicates from each of the temperature treatments were kept at the control temperature, while the other half was exposed to a new episode of increased temperature (target maximum temperature 29.0°C). The species' thermotolerance is considered to be 0°C–26°C (Jungbluth and Lehmann 1976), which is identical to the temperature range in seven Norwegian rivers with freshwater pearl mussels observed over a period of 3 years (Larsen 2012). Water temperatures in River Etna is logged approximately 10 km upstream the location where the parent mussels were collected. Summer temperature (July and August) in River Etna between 2015 and 2022 varied between 5.4°C and 23.6°C (Appendix S1). Summer temperature in River Etna exceeded 23°C in only 3 out of 372 days (data for July and August for 5 years) (Appendix S1). Consequently, our increased temperature treatments of 23°C and 26.0°C probably reflect temperature extremes experienced by Norwegian populations, whereas 29.0°C is likely to exceed the maximum water temperatures in Norwegian freshwater pearl mussel rivers. Such temperatures may however be reached locally in shallow water, during periods of low water flow and heat waves, and those events will continue to increase in frequency with the ongoing global warming.

**FIGURE 2:**
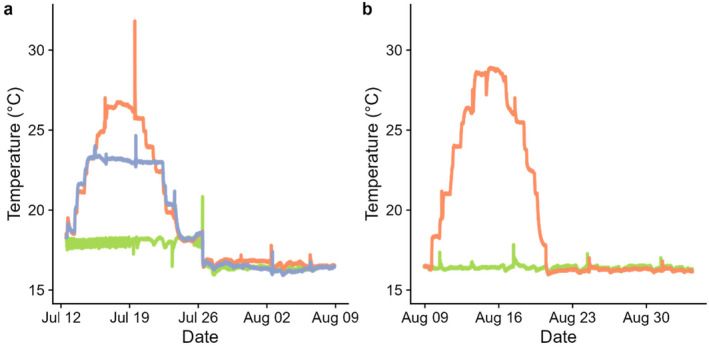
Experimental exposure of juvenile FPM to two exposures of increased water temperature. (a) Mussels were exposed to three treatments (green: control, blue: mid temperature, red: high temperature) in the first exposure. (b) Half of the mussels from each treatment were exposed to a second round of increased water temperature (red line), while the other half was kept at control temperature (green line).

To obtain the target water temperatures, we placed the boxes with mussels into channels (440 × 40 cm) with temperature‐controlled water. Water temperature in the channels was regulated by heating elements controlled by thermostats. Temperatures were adjusted upwards and downwards by respectively two and three degrees per day in both the first and second part of the experiment (Figure 2). A warming rate of three degrees per day is well within the rates observed in Norwegian rivers (Larsen 2012). To record the temperatures that the mussels experienced, one box with water (that was identical to the boxes with mussels) and a temperature logger was placed in each channel. The boxes with temperature loggers were moved from the experimental room to the feeding room in the same manner as the boxes that contained mussels. During feeding, water in the boxes with temperature loggers was changed in the same manner as in the boxes with mussels. Temperature logger data showed a short peak in temperature on one of the feeding days, that exceeded the target temperatures for the mid temperature and high temperature treatments by several degrees (Figure 2). It is unknown whether this was due to a mistake in the temperature adjustment of exchange‐water or a mistake in the handling of temperature loggers.

### 2.3 Data Collection

Dissolved oxygen was measured with a OxyMeter (WTW, Oxi 3310) in one box (containing mussels) per treatment, three times a week before and after feeding. Dissolved oxygen was negatively related to water temperature, and levels of dissolved oxygen under lower range temperatures were higher after feeding than before (Appendix S2).

We recorded mortality two times a week by individually inspecting mussels under a stereo microscope (Wild M3 Stereo Microscope), following standard procedures at the FPM rearing station at Austevoll. Dead mussels were identified visually. Mussels were always kept in sufficient water, and time under the microscope was kept to a minimum to avoid heat stress.

We measured the length of mussels before and after the experiment to compare growth between the treatments. We collected a random selection of 80 individuals before the start of the experiment and all surviving mussels at the end of the experiment. Mussels were stored in ethanol until measurement. The shell length (between the anterior and the posterior shell parts) of the mussels were measured under a stereo microscope (SZX10, Olympus, Hamburg, Germany).

### 2.4 Statistical Analysis

We used a linear mixed model to test for an effect of temperature treatment on growth during the experiment. The response variable in the model was body length at the end of the experiment and explanatory variables were temperature treatments during the first exposure (control, mid temperature, high temperature) and second exposure (control, high temperature) of the experiment. Replicate (box number) was included as a random factor. The model was fitted with an interaction between first and second exposure of temperature treatment, to test if previous exposure to increased temperatures affected the effect of the second exposure to high temperatures.

## 3 Results

### 3.1 Mortality

Mortality was very low in all treatments and not affected by temperature. Median mortality per experimental box ranged between 0 and 2 out of 30 individuals (0% to 7%) in the different treatments (Table 1). The total number of juvenile mussels that died per treatment ranged from 4 to 14 out of 300 individuals. Seven out of fourteen individuals that died in the mid temperature treatment (with control temperature in second exposure) were in the only box where fungal infection was observed during the experiment.

**TABLE 1:** Median number of juveniles per box out of 30 individuals (and total number out of 300 individuals) that over the course of the experiment died in boxes assigned to the initial three temperature treatments (control, mid and high temperature), without and with a second exposure to high temperatures.

No. dead	Control	Mid temperature	High temperature
Without second exposure	1 (7)	2 (14)	1 (6)
With second exposure	2 (5)	1 (4)	0 (6)

### 3.2 Growth

Juvenile mussels almost doubled their length from 0.56 ± 0.04 mm (mean ± SD) to 1.02 ± 0.10 mm (mean across treatments) during the experiment (Figure 1; Appendix S3). Temperature during the first exposure significantly affected growth (*χ* ^2^ = 124.9, df = 2, *p* < 0.001), with slightly higher growth in the high temperature treatment (92% growth) than in the mid (80%) and control temperature treatment (79%). Also, temperature during the second exposure significantly affected growth (*χ* ^2^ = 5.6, df = 1, *p* = 0.018), with slightly higher growth in the high temperature treatment than in the control treatment (Figure 1). The effect of the second temperature exposure on growth did not differ between the three treatments of the first exposure (*χ* ^2^ = 2.2, df = 2, *p* = 0.331). The proportion of individuals with low growth rates (below two SD of mean length at the end of the experiment) was 4% in the control treatment and 2% in the mid and high temperature treatment of the first exposure.

## 4 Discussion

Continuous exposure to temperatures above the species' known thermotolerance range for up to 6 days, did not increase the mortality of juvenile freshwater pearl mussels. Moreover, survival was consistently high, irrespective of temperature or previous exposure to heat stress. Hence, the short‐term survival of the juvenile mussels that were subjected to an extreme temperature (29°C) did not depend on thermotolerance acquired through acclimation from previous exposure to heat stress. Also, there were no signs that repeated exposure to extreme temperatures had an accumulative negative effect on survival. Taken together, our results suggest that juvenile mussels can survive repeated periods of heat stress that are considered extreme for a period of at least a few days when other environmental conditions are favourable.

The general high survival of mussels may result from that the experimental conditions followed captive breeding protocols optimised for high survival, apart from the high temperature treatments. The lack of impact of extreme temperatures on survival may therefore result from that the experimental mussels, in contrast to natural conditions, were not buried in substratum, that may normally reduce the availability of dissolved oxygen. Thereby, decreasing the ecological realism in the experiment.

In nature, poor riverbed substratum is the largest threat to the species and is considered to explain the lack of recruitment in many populations, where the availability of dissolved oxygen can be reduced by over 30% (Hastie, Boon, and Young 2000; Geist and Auerswald 2007). High water temperatures lead to further reduction in oxygen in the substrate, given the lower concentration of dissolved oxygen in the free water (Geist and Auerswald 2007). In natural rivers, periods with high water temperature are likely to occur at low water discharge, which may further reduce the oxygenation of the substratum (Quinlan et al. 2015). At the same time juvenile mussels may adjust their burrowing behaviour to move to more favourable conditions in the substratum when under stress (Hyvärinen et al. 2021).

In contrast, our set‐up with low densities of mussels exposed to free water did not test for the combined effect of high water temperatures and low O_2_‐levels. Levels of dissolved oxygen in all treatments were higher than those found to be critical for 1‐year old juvenile freshwater pearl mussels in a recent experiment (Hyvärinen, Sjönberg, Marjomäki, and Taskinen 2022). Extreme climate‐events may cause juvenile mussel mortality in the wild, depending on riverbed substratum quality (Hyvärinen, Sjönberg, Marjomäki, and Taskinen 2022). That may explain why we did not observe increased mortality, while previous work has found a negative relationship between temperature and recruitment (Gosselin et al. 2023). The absence of hypoxia in our experiment may also explain that we observed high survival at temperatures that were significantly above the previously reported range (Jungbluth and Lehmann 1976). Experimental data on both acute and long‐term thermotolerance limits (CTMax and CTMin) of the species would be highly valuable but are currently lacking. Furthermore it is possible that survival or recruitment may be affected by extreme temperatures at stages that were not included in the present study, such as sperm survival, female fecundity, timing of larvae release, glochidia mortality, development during parasitic stage and other stages during juvenile development in the substrate.

Our study tested acute and short‐term effects of increased water temperatures, and we did not follow the growth and survival of the mussels at later life‐stages. The extreme temperatures could also have caused alterations in fitness‐related phenotypic traits that we did not measure. Furthermore, it is well known that the environmental conditions an organism experiences in early life stages can have large effects on the phenotypes and even survival in later life stages (Lindström 1999; O'Connor et al. 2014). Therefore, we cannot exclude the possibility that our temperature treatments could have had an effect on the phenotypes or survival of the mussels at some later life‐stages. Juvenile mussels grew more at higher temperatures, even in the highest temperature treatment. This is in line with previous findings from a field experiment on the growth of juvenile freshwater pearl mussels (Cerna et al. 2018). Such increases in growth rate may affect later survival both positively and negatively.

Our study provides an experimental test of how extreme‐climatic events may affect juvenile freshwater pearl mussel survival. Future work is needed to test how survival is affected by high water temperatures at other life‐stages, over longer time periods and in the presence of multiple stressors.

## Author Contributions

**Sebastian Wacker:** conceptualization (equal), formal analysis (lead), funding acquisition (equal), investigation (equal), methodology (equal), project administration (equal), writing – original draft (lead). **Katrine Åmdal Sundt:** investigation (equal), methodology (equal), writing – review and editing (equal). **Jon Hamner Mageroy:** methodology (equal), writing – review and editing (equal). **Bjørn Mejdell Larsen:** methodology (equal), writing – review and editing (equal). **Chavindi Sophie Hagen:** investigation (equal), methodology (equal), writing – review and editing (equal). **Torill Horvli:** investigation (equal), methodology (equal), writing – review and editing (equal). **Grethe Robertsen:** conceptualization (equal), funding acquisition (equal), investigation (equal), methodology (equal), project administration (equal), writing – review and editing (lead).

## Conflicts of Interest

The authors declare no conflicts of interest.

## Supporting information


Appendix S1.

Appendix S2.

Appendix S3.

## Data Availability

Data are provided as supplementary files and will be archived at Dryad upon acceptance of the manuscript.
