# Correction to “Resilience of Juvenile Freshwater Pearl Mussels to Thermal Stress”

**DOI:** 10.1002/ece3.70679

**Published:** 2025-01-19

Wacker, S., K. Sundt, J. Mageroy, et al. 2024. “Resilience of Juvenile Freshwater Pearl Mussels to Thermal Stress.” *Ecology and Evolution* 14: e70456. https://doi.org/10.1002/ece3.70456.

Due to a production error, Appendix Figures 1, 2, and 3 were erroneously removed. The previously published Supporting Information has been removed and is now available via the link in the Data Availability Statement. The online version of this article has been corrected accordingly.

We apologize for this error.

